# Design, Synthesis, and Evaluation of Dihydrobenzo[*cd*]indole-6-sulfonamide as TNF-α Inhibitors

**DOI:** 10.3389/fchem.2018.00098

**Published:** 2018-04-04

**Authors:** Xiaobing Deng, Xiaoling Zhang, Bo Tang, Hongbo Liu, Qi Shen, Ying Liu, Luhua Lai

**Affiliations:** ^1^Peking–Tsinghua Center for Life Sciences, Peking University, Beijing, China; ^2^Center for Quantitative Biology, Academy for Advanced Interdisciplinary Studies, Peking University, Beijing, China; ^3^BNLMS, State Key Laboratory for Structural Chemistry of Unstable and Stable Species, College of Chemistry and Molecular Engineering, Peking University, Beijing, China

**Keywords:** TNF-α inhibitor, dihydrobenzo[*cd*]indole-6-sulfonamide, virtual screening, synthesis, structure activity analysis

## Abstract

Tumor necrosis factor-α (TNF-α) plays a pivotal role in inflammatory response. Dysregulation of TNF can lead to a variety of disastrous pathological effects, including auto-inflammatory diseases. Antibodies that directly targeting TNF-α have been proven effective in suppressing symptoms of these disorders. Compared to protein drugs, small molecule drugs are normally orally available and less expensive. Till now, peptide and small molecule TNF-α inhibitors are still in the early stage of development, and much more efforts should be made. In a previously study, we reported a TNF-α inhibitor, **EJMC-1** with modest activity. Here, we optimized this compound by shape screen and rational design. In the first round, we screened commercial compound library for **EJMC-1** analogs based on shape similarity. Out of the 68 compounds tested, 20 compounds showed better binding affinity than **EJMC-1** in the SPR competitive binding assay. These 20 compounds were tested in cell assay and the most potent compound was 2-oxo-N-phenyl-1,2-dihydrobenzo[*cd*]indole-6-sulfonamide (**S10**) with an IC_50_ of 14 μM, which was 2.2-fold stronger than **EJMC-1**. Based on the docking analysis of **S10** and **EJMC-1** binding with TNF-α, in the second round, we designed **S10** analogs, purchased seven of them, and synthesized seven new compounds. The best compound, **4e** showed an IC_50_-value of 3 μM in cell assay, which was 14-fold stronger than **EJMC-1**. **4e** was among the most potent TNF-α organic compound inhibitors reported so far. Our study demonstrated that 2-oxo-N-phenyl-1,2-dihydrobenzo[*cd*]indole-6-sulfonamide analogs could be developed as potent TNF-α inhibitors. **4e** can be further optimized for its activity and properties. Our study provides insights into designing small molecule inhibitors directly targeting TNF-α and for protein–protein interaction inhibitor design.

## Introduction

Tumor necrosis factor-α (TNF-α), an important cytokine mediator involved in inflammatory responses, is commonly used as a marker for many inflammatory disorders (Wajant et al., 2003). Antibodies that directly targeting TNF-α have achieved success in the treatment of inflammatory disorders such as rheumatoid arthritis, Crohn's disease, and ulcerative colitis (Bongartz et al., 2005; Jacobi et al., 2006). However, these biologics possess the possibility to cause anti-antibody immune responses and weaken the immune system to opportunistic infections (Scheinfeld, 2004; Ai et al., 2015).

Thus, developing inhibitors to block TNF-α is still of great importance. Zhu et al. have reported several rationally designed proteins that directly bound to TNF-α. They grafted three key residues from a virus viral 2L protein to a *de novo* designed small protein DS119, and then optimized their residues at the interface, which provided some small proteins that bind TNF-α with sub-micromolar affinities (Zhu et al., 2016). Other than small proteins, bicyclic peptides and helical peptides were also designed as peptidic antagonists of TNF-α (Lian et al., 2013; Zhang et al., 2013).

**Figure 1:**
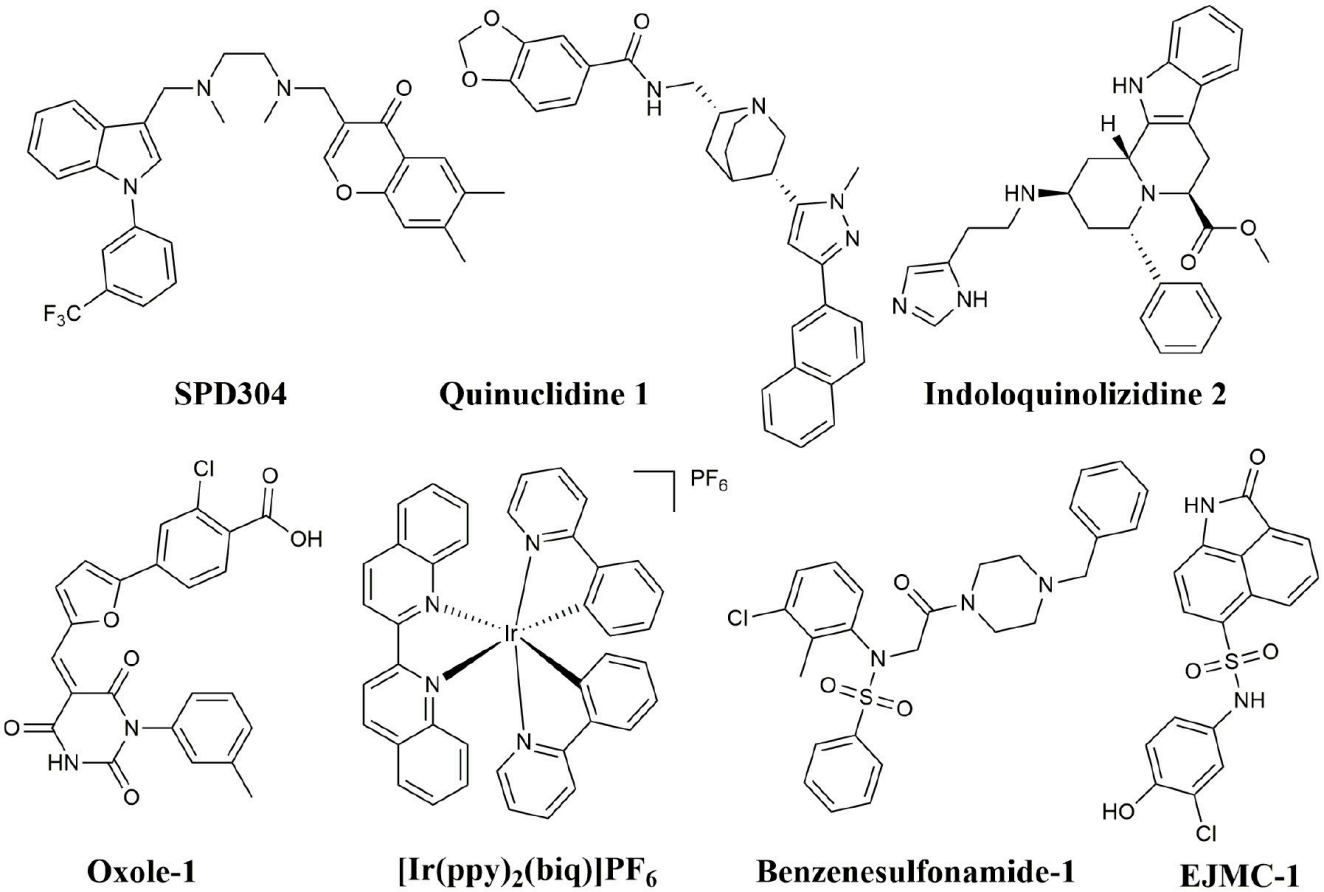
Structures of small molecule inhibitors of TNF-α.

In addition to peptide inhibitors, small molecular inhibitors that directly targeting TNF-α have also been discovered (Leung et al., 2012; Davis and Colangelo, 2013; Shen et al., 2014). Suramin was thought to be the first small compound inhibitor that directly disrupts the interactions between TNF-α and its receptor (TNFR) (Grazioli et al., 1992). But its potency was too low to be used in clinic (Alzani et al., 1993). No breakthrough was made until 2005, when SPD304 was reported as the first potent small molecule inhibitor that directly targeting TNF-α, with an IC_50_ of 22 μM by ELISA. And the co-crystal structure of SPD304 in complex with TNF-α dimer was solved (He et al., 2005). However, as the 3-alkylindole moiety of SPD304 can be metabolized by cytochrome P450s to produce toxic electrophilic intermediates, its further applications *in vivo* is limited (Sun and Yost, 2008). After that, several novel TNF-α inhibitors were discovered using structure-based virtual screening (VS) of different chemical libraries. Chan et al. identified two compounds using high-throughput ligand-docking-based VS (Figure 1, **quinuclidine 1** and **indoloquinolizidine 2**), and their experimental tests showed that **quinuclidine 1** is more effective than **indoloquinolizidine 2** in inhibition of TNF-α induced NF-κB signaling in HepG2 cells, with IC_50_-values of 5 and >30 μM, respectively (Chan et al., 2010). Choi and colleagues discovered a series of pyrimidine-2,4,6-trione derivatives from a 240,000-compound library. The best compound (Figure 1, **Oxole-1**) showed 64% inhibition at 10 μM (Choi et al., 2010). Leung et al. reported a novel iridium(III)-based direct inhibitor of TNF-α (Figure 1, **[Ir(ppy)_2_(biq)]PF_6_**; Leung et al., 2012). Mouhsine et al. used combined *in silico/in vitro/in vivo* screening approaches to identify orally available TNF-α inhibitors with IC_50_ of 10 μM (Figure 1, **Benzenesulfonamide-1**; Mouhsine et al., 2017). Other efforts to develop TNF-α inhibitors were also reported (Mancini et al., 1999; Buller et al., 2009; Leung et al., 2011; Hu et al., 2012; Alexiou et al., 2014; Ma et al., 2014; Kang et al., 2016). However, due to the low potency and high cytotoxicity, small molecule TNF-α inhibitors still have a long way to go for clinical applications (Davis and Colangelo, 2013). Highly active TNF-α inhibitors with novel chemical structures need to be developed. In a previous study, we have discovered a compound (Figure 1, **EJMC-1**) that directly bound TNF-α (Shen et al., 2014). The scaffold of the compound, 2-oxo-N-phenyl-1,2-dihydrobenzo[*cd*]indole-6-sulfonamide, has been reported as inhibitors of West Nile virus (Gu et al., 2006), RORγ inhibitors (Zhang et al., 2014), and BET bromodomain inhibitors (Xue et al., 2016; Mouhsine et al., 2017). Considering the good druggability of this scaffold, its analogs may be valuable for developing potent TNF-α inhibitors. In the present study, we used the scaffold of compound **EJMC-1** to perform similarity-based virtual screen and experimental testing. Top-ranking compounds were first tested for their abilities to reduce TNF-α binding with TNFR using surface plasmon resonance (SPR). Then the cell-based NF-κB reporter gene assay was used to test the activities of the compounds to reduce TNF-α induced signaling. New compounds were further designed, synthesized, and tested. The structure-activity relationship of these compounds was analyzed.

## Materials and methods

### General information

HEK293T cells were received as a gift from Professor Jincai Luo (Peking University, China). The extracellular domain of the TNF receptor 1 (TNFR1-ECD) was purchased from R&D Systems. The selected compounds were purchased from the SPECS database with purity higher than 90% and for most compounds >95% (confirmed by the supplier, using NMR or LC-MS data available through the website). Other biochemistry reagents were from Sigma Aldrich unless indicated otherwise. The organic reagents and solvents were commercially available and purified according to conventional methods. All reactions were monitored by thin layer chromatography (TLC), using silica gel 60 F-254 aluminum sheets and UV light (254 and 366 nm) for detection. All title compounds gave satisfactory ^1^H NMR, ^13^C NMR, and mass spectrometry analyses. The ^1^H NMR and ^13^C NMR spectra were measured on a Bruker-400 M spectrometer using TMS as internal standard. High resolution mass spectra were recorded on a Bruker Apex IV FTMS mass spectrometer using ESI (electrospray ionization).

### Synthesis

#### Benzo[*cd*]indol-2(*1H*)-one 2

**2** was prepared based on the adoption of method by Kamal et al. (2012). Napthalic anhydride (1.98 g, 10 mmol), hydroxylamine hydrochloride (0.69 mg, 10 mmol), and dry pyridine (5 ml) were added to dried three-necked flask. Heating was discontinued after reflux for 1 h, than benzenesulfonyl chloride (5 g) was added portion wise to cause controlled boiling. Finally, heating was resumed for 1 h, and the hot mixture was poured into water (30 ml). The crystalline precipitate was collected, washed with 0.5 N NaOH and water. The crystals were boiled with water (15 ml) and ethanol (5 ml) containing sodium hydroxide (5 g) for 2 h, during the second of which, ethanol was allowed to distill out. The solution was acidified with concentrated hydrochloric acid (3 ml), carbon dioxide being evolved and yellow crystals deposited. Next day, the crystals were washed, and dried to give light yellow needles (1.25 g, 74%). Mp175–179°C; ^1^H NMR (DMSO, 300 MHz, DMSO-*d*_6_) δ 8.05 (d, 1H, *J* = 6.7 Hz), 8.01 (d, 1H, *J* = 8.3 Hz), 7.75–7.70 (m, 1H), 7.53 (d, 1H, *J* = 8.3 Hz), 7.40 (dd, 1H, *J* = 7.5, 6.7 Hz), 6.94 (d, 1H, *J* = 6.7 Hz).

#### 2-oxo-1,2-dihydrobenzo[*cd*]indole-6-sulfonyl chloride 3

**3** was prepared based on the adoption of method by Talukdar et al. (2010). Chlorosulfonic acid (3.2 ml) was added slowly to **2** (1.0 g, 5.9 mmol). The reaction mixture was stirred at 0°C for 1 h and at room temperature for 2 h. The mixture was then poured into ice water (20 ml). The precipitate was washed with water (2 × 10 ml) and dried to give product as yellow solid (0.66 g, 38%). Used without further purification.

### General procedure for N-substituted 2-oxo-N-phenyl-1,2-dihydrobenzo[*cd*]ind-ole-6-sulfonamides 4

A mixture of 2-oxo-1,2-dihydrobenzo[*cd*]indole-6-sulfonyl chloride (100 mg, 0.37 mmol), 0.37 mmol aniline, 0.4 ml Et_3_N, 20 mg DMAP was dissolved in 5 ml DMF, the reaction mixture was stirred at room temperature, the reaction was detected by TLC, after the reaction was finished, extracted with 50 ml ethyl acetate and 20 ml water, washed with water 20 ml three times, then 20 ml saturated NH_4_Cl aqueous, 20 ml brine. The organic layer was dried by Na_2_SO_4_, and the solvent was removed *in vacuo*. The residue was purified by Column chromatography.

#### N-(5-aminonaphthalen-1-yl)-2-oxo-1,2-dihydrobenzo[*cd*]indole-6-sulfonamide 4a

Seventy-six milligrams, yield 54%. ^1^H NMR (400 MHz, DMSO-*d*_6_) δ 5.66 (s, 2H), 6.52 (d, *J* = 7.5 Hz, 1H), 6.88 (t, *J* = 8.1 Hz, 1H), 6.92 (d, *J* = 7.6 Hz, 1H), 7.04 (d, *J* = 8.4 Hz, 1H), 7.11 (d, *J* = 7.3 Hz, 1H), 7.18 (t, *J* = 7.9 Hz, 1H), 7.87 (dd, *J* = 7.9, 4.0 Hz, 3H), 8.07 (d, *J* = 7.0 Hz, 1H), 8.65 (d, *J* = 8.4 Hz, 1H), 10.18 (s, 1H), 11.07 (s, 1H). ^13^C NMR (101 MHz, DMSO-*d*_6_) δ 168.70, 144.73, 142.65, 132.68, 132.01, 130.51, 130.23, 129.48, 128.50, 126.64, 126.46, 125.92, 124.71, 124.56, 123.29, 122.84, 122.76, 121.03, 110.30, 107.63, 104.54. HRMS (ESI): calcd for C_21_H_16_N_3_O_3_S, [(M+H)^+^], 391.0912, found 390.0896.

#### N-(3-aminonaphthalen-2-yl)-2-oxo-1,2-dihydrobenzo[*cd*]indole-6-sulfonamide 4b

Thirty-four milligrams, yield 26%. ^1^H NMR (400 MHz, DMSO-*d*_6_) δ 1.20–1.29 (m, 2H), 4.67–5.36 (m, 2H), 6.79 (s, 1H), 6.95 (d, *J* = 7.6 Hz, 1H), 7.05 (ddd, *J* = 8.1, 6.7, 1.2 Hz, 1H), 7.22 (ddd, *J* = 8.2, 6.8, 1.3 Hz, 1H), 7.35 (s, 1H), 7.41 (d, *J* = 8.2 Hz, 1H), 7.46 (d, *J* = 8.2 Hz, 1H), 7.85 (dd, *J* = 8.4, 7.0 Hz, 1H), 7.95 (d, *J* = 7.6 Hz, 1H), 8.08 (d, *J* = 7.0 Hz, 1H), 8.65 (d, *J* = 8.4 Hz, 1H), 11.12 (s, 1H). ^13^C NMR (101 MHz, DMSO-*d*_6_) δ 169.10, 143.82, 142.21, 132.68, 132.21, 130.81, 130.01, 129.31, 128.65, 126.66, 126.45, 125.87, 124.63, 124.53, 123.39, 122.81, 122.77, 121.03, 110.60, 106.63, 103.59. HRMS (ESI): calcd for C_21_H_16_N_3_O_3_S, [(M+H)^+^], 390.0912, found 390.0896.

#### 2-oxo-N-(1,2,3,4-tetrahydronaphthalen-1-yl)-1,2-dihydrobenzo[*cd*]indole-6-sulfonamide 4c

Sixty-eight milligrams, yield 48%. ^1^H NMR (400 MHz, DMSO-*d*_6_) δ 1.31–1.42 (m, 2H), 1.55–1.74 (m, 2H), 2.55–2.70 (m, 2H), 4.33 (dd, *J* = 9.7, 5.3 Hz, 1H), 6.93–6.97 (m, 2H), 7.02 (s, 1H), 7.08 (d, *J* = 7.4 Hz, 2H), 7.90–7.95 (m, 1H), 8.14 (t, *J* = 6.9 Hz, 2H), 8.35 (d, *J* = 8.4 Hz, 1H), 8.72 (d, *J* = 8.4 Hz, 1H), 11.16 (s, 1H). ^13^C NMR (101 MHz, DMSO-*d*_6_) δ 169.31, 142.98, 137.57, 136.86, 132.65, 130.89, 130.37, 130.12, 129.17, 128.95, 127.41, 127.35, 126.67, 126.10, 125.31, 124.82, 105.17, 51.51, 30.68, 28.87, 19.68. HRMS (ESI): calcd for C_42_H_36_N_4_NaO_6_S_2_, [(2M+Na)^+^], 779.1974, found 799.1939.

#### N-(naphthalen-1-ylmethyl)-2-oxo-1,2-dihydrobenzo[*cd*]indole-6-sulfonamide 4d

Fifteen milligrams, yield 10%. ^1^H NMR (400 MHz, DMSO-*d*_6_) δ 4.43 (d, *J* = 5.7 Hz, 2H), 6.94 (d, *J* = 7.5 Hz, 1H), 7.25–7.36 (m, 3H), 7.41 (ddd, *J* = 8.1, 6.8, 1.2 Hz, 1H), 7.72–7.76 (m, 1H), 7.82 (d, *J* = 8.1 Hz, 1H), 7.85–7.90 (m, 2H), 7.98 (d, *J* = 7.5 Hz, 1H), 8.08 (d, *J* = 7.0 Hz, 1H), 8.40 (t, *J* = 5.9 Hz, 1H), 8.65 (d, *J* = 8.3 Hz, 1H), 11.10 (s, 1H). ^13^C NMR (101 MHz, DMSO-*d*_6_) δ 169.29, 133.54, 132.94, 132.84, 131.08, 130.74, 130.04, 128.93, 128.69, 128.47, 127.29, 126.52, 126.33, 126.07, 125.47, 125.13, 124.74, 123.89, 104.98, 44.73. HRMS (ESI): calcd for C_44_H_33_N_4_O_6_S_2_, [(2M+H)^+^], 777.1842, found 777.1804.

#### N-(1H-indol-6-yl)-2-oxo-1,2-dihydrobenzo[*cd*]indole-6-sulfonamide 4e

Ninety-one milligrams, yield 68%. ^1^H NMR (300 MHz, DMSO-*d*_6_) δ 6.21–6.27 (m, 1H), 6.67 (dd, *J* = 8.4, 2.0 Hz, 1H), 6.95 (d, *J* = 7.7 Hz, 1H), 7.05 (d, *J* = 1.7 Hz, 1H), 7.17–7.22 (m, 1H), 7.27 (d, *J* = 8.5 Hz, 1H), 7.91 (dd, *J* = 8.4, 7.0 Hz, 1H), 7.97 (d, *J* = 7.6 Hz, 1H), 8.08 (d, *J* = 6.9 Hz, 1H), 8.74 (d, *J* = 8.3 Hz, 1H), 10.20 (s, 1H), 10.91 (s, 1H), 11.11 (s, 1H). ^13^C NMR (101 MHz, DMSO-*d*_6_) δ 169.15, 143.24, 136.18, 133.83, 131.41, 130.96, 129.77, 128.07, 127.28, 126.38, 125.87, 125.29, 124.90, 120.64, 114.44, 105.02, 104.61, 101.32. HRMS (ESI): calcd for C_38_H_27_N_6_O_6_S_2_, [(2M+H)^+^], 727.1433, found 727.1428.

#### N-(3-(1-methyl-1H-pyrazol-4-yl)phenyl)-2-oxo-1,2-dihydrobenzo[*cd*]indole-6-sulfonamide 4f

Seventy-six milligrams, yield 51%. ^1^H NMR (300 MHz, DMSO-*d*_6_) δ 3.84 (s, 3H), 6.80 (dt, *J* = 5.4, 2.8 Hz, 1H), 7.04 (d, *J* = 7.6 Hz, 1H), 7.09 – 7.13 (m, 2H), 7.19 (s, 1H), 7.64 (s, 1H), 7.90 – 7.95 (m, 1H), 7.98 (s, 1H), 8.09 (d, *J* = 7.0 Hz, 1H), 8.19 (d, *J* = 7.7 Hz, 1H), 8.72 (d, *J* = 8.3 Hz, 1H), 10.59 (s, 1H), 11.16 (s, 1H). ^13^C NMR (101 MHz, DMSO-*d*_6_) δ 169.25, 143.33, 136.18, 133.83, 133.56, 131.41, 130.96, 130.51, 129.77, 128.07, 127.28, 126.38, 125.87, 125.29, 124.90, 124.64, 119.44, 117.82, 117.61, 114.32, 40.51. HRMS (ESI): calcd for C_21_H_17_N_4_O_3_S, [(M+H)^+^], 405.1021, found 405.1086.

#### 6-((1H-benzo[d]imidazol-1-yl)sulfonyl)benzo[*cd*]indol-2(1H)-one 4g

Eighty milligrams, yield 62%. ^1^H NMR (400 MHz, DMSO-*d*_6_) δ 7.16 (d, *J* = 7.8 Hz, 1H), 7.37-7.31 (m, 2H), 7.69–7.75 (m, 1H), 7.82 (dt, *J* = 8.3, 0.9 Hz, 1H), 7.93–8.03 (m, 1H), 8.12 (d, *J* = 7.0 Hz, 1H), 8.71 (d, *J* = 7.8 Hz, 1H), 8.76 (d, *J* = 8.4 Hz, 1H), 9.19 (s, 1H), 11.35 (s, 1H). ^13^C NMR (101 MHz, DMSO-*d*_6_) δ 168.46, 145.77, 143.47, 142.38, 136.22, 132.11, 129.83, 127.72, 127.05, 126.04, 125.57, 125.46, 124.71, 123.50, 122.77, 120.65, 112.18, 104.85. HRMS (ESI): calcd for C_36_H_23_N_6_O_6_S_2_, [(2M+H)^+^], 699.1120, found 699.1135.

### Competitive binding assay using SPR

Binding interactions between TNF-α and TNFR1-ECD in the presence/absence of small molecule inhibitors were examined on the SPR-based Biacore T200 instrument (GE Healthcare). TNFR1-ECD was immobilized on a CM5 sensor chip using standard amine-coupling at 25°C with 1X running buffer PBS-P (GE Healthcare). A reference flow cell was activated and blocked in the absence of TNFR1-ECD. All experiments were performed in phosphate-buffered saline (PBS)-EP buffer (10 mM NaH_2_PO_4_/Na_2_HPO_4_, 150 mM NaCl, 3.7 mM EDTA, 0.05% surfactant P20, pH 7.4) at 25°C with a flow rate of 50 μl/min. A final concentration of 20 nM TNF-α was mixed with each compound at various concentrations (as indicated in section Results) in PBS-EP and the mixture was injected. Equal amounts of TNF-α mixed with PBS-EP were used as a control. Regeneration was achieved by extended washing with glycine hydrochloride buffer (10 mM Glycine-HCl, pH 2.1) after each sample injection.

### Cell based NF-κB reporter assay

The cellular assay were carried out as described previously (Zhang et al., 2013). HEK293T cells were grown to 70% confluence in 6 cm dish at 37°C in Dulbecco's modified Eagle's medium supplemented with 10% fetal bovine serum (FBS; Gibco), then transfected with purified plasmids 0.6 μg pGL4.32 (luc2P/NF-κB-RE/Hygro plasmid) and 0.4 μg pGL4.74 (hRluc/TK) with ViaFect transfection reagent (Promega). After 24 h, the transfected cells were seeded in 96-wells plate, 40,000 cells per well. Twelve hours later, 100 μL pre-incubated mixture of TNF-α and small molecules was added to stimulate the cells for 6 h and the luciferase assays were carried out using the Dual-Glo Luciferase Assay System (Promega) with a BioTek synergy 4 Multi-Mode Microplate Reader. The final concentration of TNF-α in each well was 10 ng/ml. Equal amounts of TNF-α without small molecular were added to the cells as a negative control to calculate the percentage of activity inhibition.

### Similarity-based virtual screen

The crystal structure of TNF-α dimer (PDB code: 2AZ5) was used for grid generation. The program Glide Standard Precise (SP) mode was used to do the molecular docking studies (Friesner et al., 2004; Halgren et al., 2004). **EJMC-1** was first docked to TNF-α dimer, and its conformation in the complex was used for Shape Screening of the SPECS library (May 2013 version for 10 mg; 197,276 compounds). The Shape Similarity indexes between each compound in the library and the reference compound were calculated. A total of 587 compounds with indexes between 0.8 and 0.99 were selected as candidates for the second round manual selection with the following selection criteria: (a) containing at least one ring which provides hydrophobic interaction; (b) containing no metal atoms; and (c) shared in multiple structures. A total of 68 compounds were purchased from SPECS for experimental testing.

### Molecular docking

The complex structure of TNF-α with SPD304 (PDB code: 2AZ5) was retrieved from the Protein Data Bank and docking was performed with maestro (Schrödinger, Inc., version 10.2). Compound **EJMC-1, S10**, and **4e** were docked into TNF-α dimer protein using Glide Docking module (Friesner et al., 2004; Halgren et al., 2004). The details of the docking workflow are listed below: (1) Protein was prepared using the “Protein Preparation Wizard” workflow. All water molecules were removed from the structure of the complex. Hydrogen atoms and charges were added during a brief relaxation. After optimizing the hydrogen bond network, the crystal structure was minimized using the OPLS_2005 force field with the maximum root mean square deviation (RMSD) value of 0.3 Å. (2) The ligand was prepared with LigPrep module in Maestro, including adding hydrogen atoms, ionizing at a pH range from 7.2 to 7.4, and producing the corresponding low-energy 3D structure. (3) Pose prediction mode of Glide Docking modules were adopted to dock the molecules into the SPD304-binding site with the default parameters. The center of the grid box was defined with SPD304. The top-ranking poses of molecule **EJMC-1, S10**, and **4e** were retained. The LigPrep mol2 format output was also docked using AutoDock Vina (Trott and Olson, 2010) with standard protocols. The computed binding free energies and structures for the top conformations were saved for post-docking analysis.

### Statistical analysis

Cell assay was repeated for three times. Statistical analysis was performed using OriginPro 9.1, data was fit by DoseResp using Origin 9.1. DoseResp was a three-parameter Hill equation. Results were expressed as mean ± *SD* (standard deviation value).

## Results and discussion

### Chemistry

**Scheme 1:**
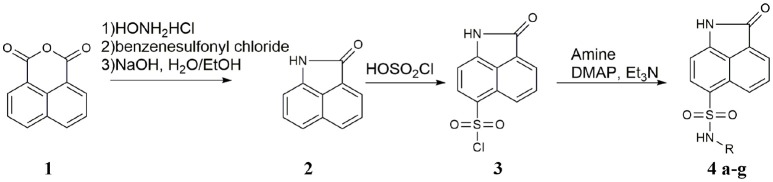
Synthesis of 2-oxo-1,2-dihydrobenzo[*cd*]indole-6-sulfonamide derivatives.

Seven derivatives of dihydrobenzo[*cd*]indole-6-sulfonamide were synthesized using a three-step synthetic route (Scheme 1) with yields between 10 and 68%. Napthalic anhydride was transformed to benzo[*cd*]indol-2(*1H*)-one by aminolysis reaction smoothly, with a yield of 74%. Then, benzo[*cd*]indol-2(*1H*)-one underwent nucleophile substitution reaction with chlorosulfonic acid to get key intermediate 2-oxo-1,2-dihydrobenzo[*cd*]indole-6-sulfonyl chloride (**3**), with a yield of 38%. Reactions of compound **3** with various amines in the presence of a catalyst system consisting of DMAP, Et_3_N, afforded **4** and derivatives in good yields. The original spectra of featured compounds shown in Supplementary Image 1.

### Compounds from similarity search of EJMC-1 block TNF-α binding to TNFR

We used compound **EJMC-1** as the reference compound for similarity search (Figure 1). The binding conformation of **EJMC-1** with TNF-α was generated using molecular docking and used in pharmacophore based shape screening over the SPECS library. Compounds with similarity index between 0.80 and 0.99 with **EJMC-1** were subjected to further manual selection. A total of 68 compounds were selected for experimental testing (Table S1). The chemical structures of these compounds fall into two classes, sulfonates and sulfonamides. The sulfonamides contain both N-aryl sulfonamides and N-alkylsulfonamides, with or without substituted aminocarbonyl group (Table S1).

**Figure 2:**
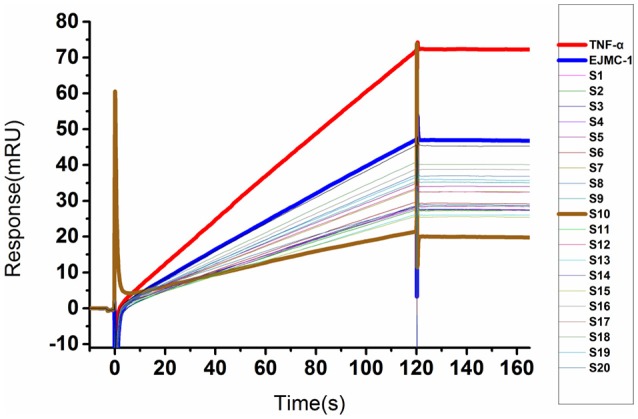
SPR competitive binding curves of compounds from shape screening of **EJMC-1**. Compounds showed competitive binding to TNF-α. The Red curve was TNF-α binding with TNFR1-ECD alone, and the other curves were TNF-α TNFR1-ECD in the presence of compounds at 100 μM. The reference compound **EJMC-1** was colored blue and the best compound in SPR assay **S10** was colored brown.

We used a SPR competitive assay to test whether these compounds can more efficiently block TNF-α and TNFR binding than **EJMC-1**. TNF-α with or without compounds flowed over the chip surface where the extracellular domain of TNFR was immobilized. At the concentration of 100 μM, 20 of the 68 compounds reduced the TNF-α binding signal compared to **EJMC-1** (Figure 2). These 20 candidates were selected for further cell-based inhibition studies. The specs ID of these 20 compounds were listed in Table S2, and the corresponding chemical structures were in supporting information. All the sulfonamide derivatives of **EJMC-1** showed competitive binding with TNF-α against TNFR1, while sulfonates could not.

### EJMC-1 analogs inhibit TNF-α induced NF-κB gene expression

**Figure 3:**
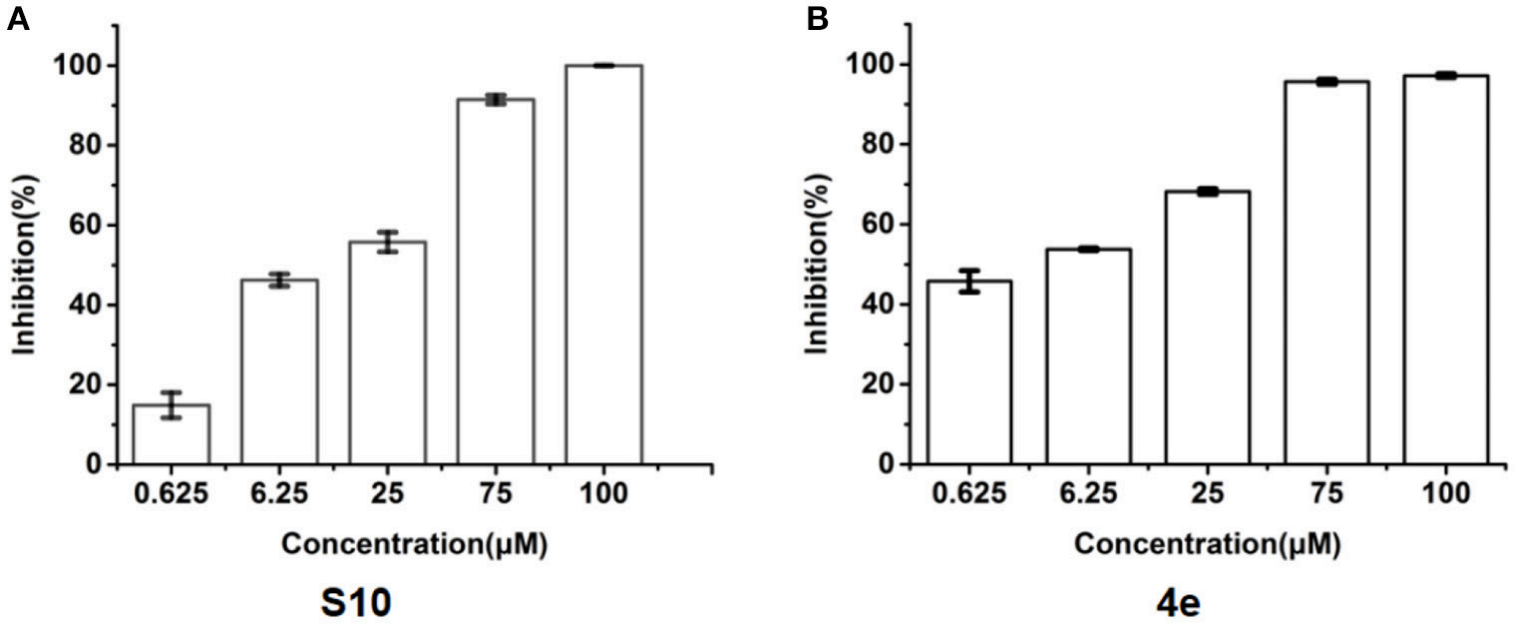
Inhibition of TNF-α induced NF-κB transcription activity. **(A)** Dose-response of compounds S10 in the cell based assay in 293T cell line. **(B)** Dose-response of compounds **4e** in the cell based assay in 293T cell line. The data was reported as means ± errors from three independent experiments.

To explore whether these compounds with enhanced abilities to reduce TNF-α binding with receptor were active under cellular environment, we used a luciferase assay to monitor their influences on NF-κB transcriptional activity. In this assay, in transfected cells, TNF-α induces NF-κB activation through TNFR1, which then drives the expression of the luciferase. The cell-level inhibitory effects of these 20 compounds were measured using the Dual-Glo Luciferase Assay System. With two dose screen, two compounds, **S3 and S10** showed better activity than **EJMC-1** (Table S2). The best compound, **S10**, suppressed NF-κB transcriptional activity dose-dependently (Figure 3A) with an IC_50_ of 19.1 ± 2.2 μM. The positive control, SPD304, displayed an IC_50_ of 6.4 ± 0.6 μM in the side-by-side experiment.

### Docking analysis and compound design

**Figure 4:**
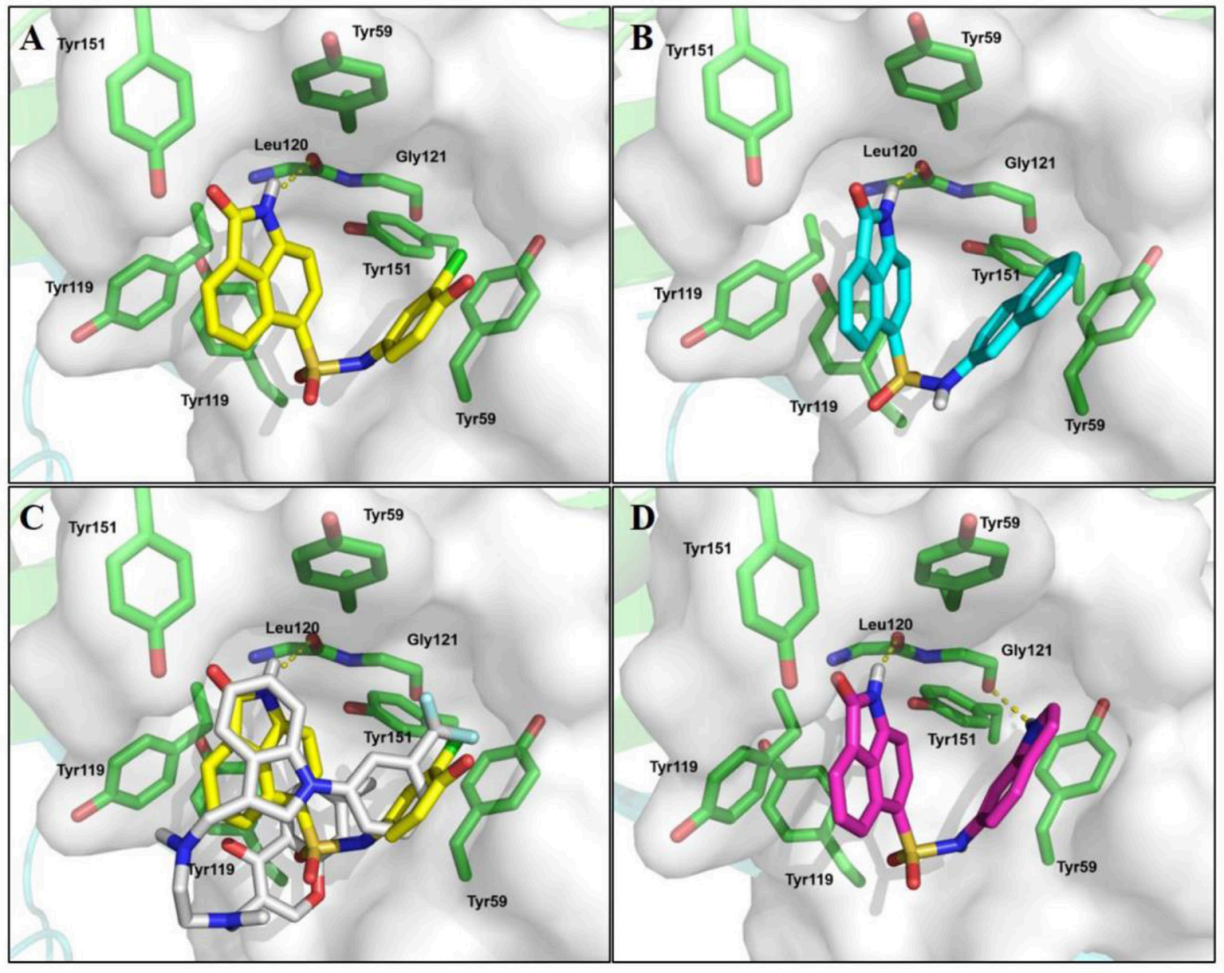
The predicted binding modes of TNF-α inhibitors. Predicted binding mode of compounds **EJMC-1** and **4e** to TNF-α. The binding site was shown as surface, the key residues were shown as sticks (green). **(A)** compound **EJMC-1** (yellow). **(B)** Compound **S10** (cyan). **(C) EJMC-1** compare to **SPD304** (gray). **(D)** compound **4e** (magenta).

Molecular docking gave clues on rational designing compounds with potential enhanced activities and understanding SAR. In the complex structure of TNF-α with SPD304, SPD304 bound to a pocket in the TNF-α dimer (He et al., 2005; Shen et al., 2014). **EJMC-1** was shown to bind with the same site (He et al., 2005; Shen et al., 2014). Both Glide and AutoDock Vina were used in the docking study. We first tested whether the binding pose of SPD304 can be reproduced. We have tried many times with different parameters, but was unable to get a binding pose that is close to that in the crystal structure (with the minimum RMSD up to 4 Å). We then used AutoDock Vina to dock SPD 304 to the TNF-α dimer and the lowest binding free energy conformation obtained was closed to its crystal conformation with a RMSD of 0.70 Å. Despite of the different binding conformations obtained for SPD304 by using two docking software, the top ranking conformations of **EJMC-1** were almost the same from the docking runs using both Glide and AutoDock Vina. These differences might due to the flexibility of SPD304, which adopted a U shape conformation, and the conformational sampling preference of the docking software. As there are no essential differences in the docking poses of the compounds other than SPD304, we used the Glide docking poses of these compounds to compare to SPD304 in the crystal structure. Compared to **EJMC-1**, **S10** had increased hydrophobic interaction with the Tyr59 residue (Figures 4A,B). In addition to the nonpolar interactions with TNF-α as in the case of SPD304, the scaffold of **EJMC-1** and **S10** provide further polar interactions, strengthening the specificity and activity (Figure 4C). As **EJMC-1** is smaller than that of SPD304 with unoccupied hydrophobic space in the pocket (Figure 4C), several analogs with larger substituted group of sulfonamide of 2-oxo-1,2-dihydrobenzo[*cd*]indole-6-sulfonamide were designed and docked to this site. Compound **4e**, with larger hydrophobic group size and additional H-bond donor, interacts favorably with TNF-α and might be more potent (Figure 4D). Based on the docking analysis, the designed analogs were purchased or synthesized for cell assay.

### Optimization of compound S10 and structure-activity analysis

**Figure 5:**
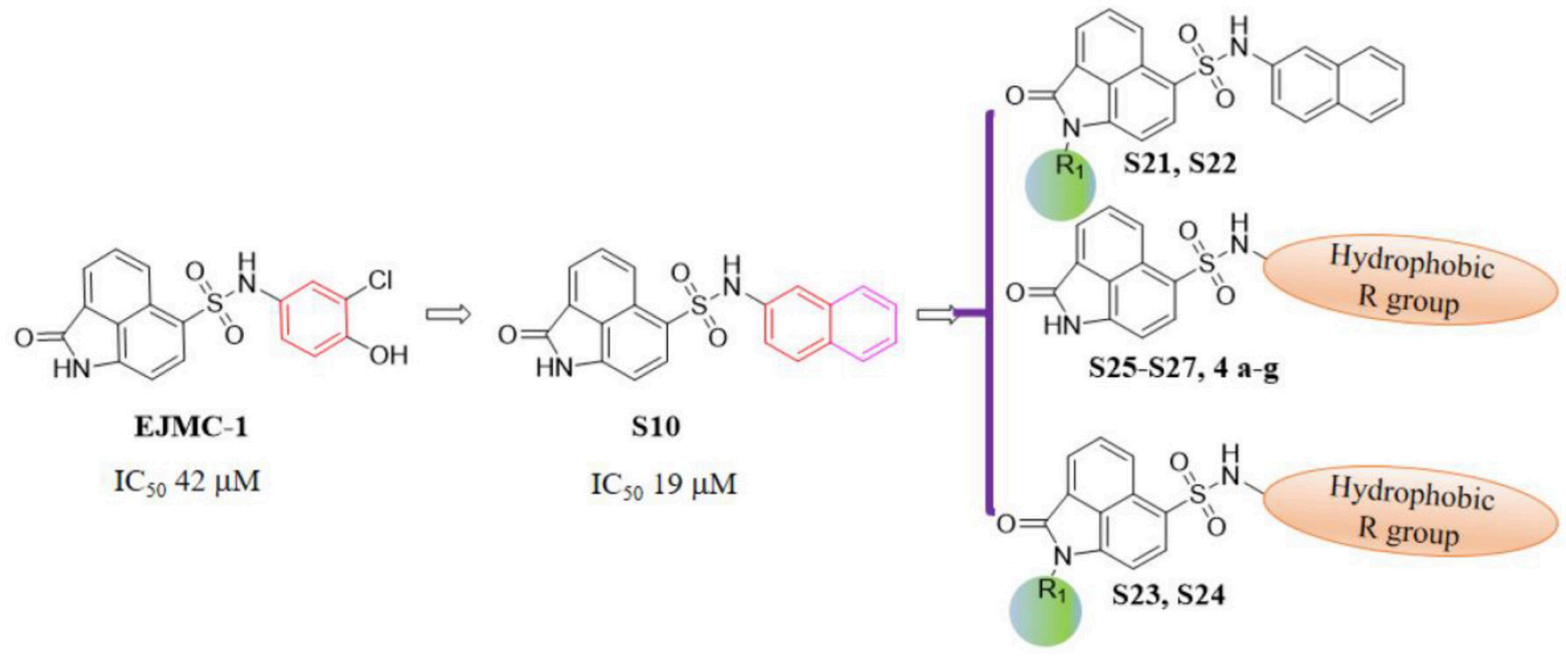
Designed TNF-α inhibitors.

As shown in the cell assay, the inhibition activity of **S10** increased about 2-fold than that of **EJMC-1**. The introduction of the naphthalene ring provides stronger hydrophobic interactions. Based on the docking analysis and increased activity of **S10**, we try to: (1) Keep naphthalene ring, changed N-substituted groups of dihydrobenzo[*cd*]indole, (2) Keep N-H of dihydrobenzo[*cd*]indole, optimize the hydrophobic R group, (3) optimize both N-substituted groups of dihydrobenzo[*cd*]indole and the hydrophobic R group (Figure 5). Seven commercially available analogs of **S10** were purchased for testing (Table 1). The SPECS ID of these seven **S10** analogues were listed in Table S3. We further synthesized seven new compounds in three steps from 1,8-Naphthalic anhydride through conventional reactions (Scheme 1 and Figure 5). All compounds passed the PAINS (pan assay interference compounds) remover, which filters out compounds that appear as frequent hitters (promiscuous compounds) in many biochemical high throughput screens (Baell and Holloway, 2010).

**Table 1:** The structure and activities of **S10** analogs.

**Compound**	**Structure**	**IC_50_ in cell assay (μM)^a^**	**Source**
**EJMC-1**	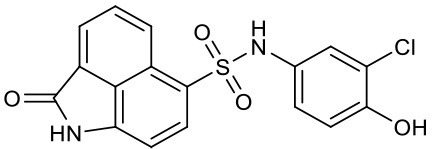	43.2 ± 2.6	SPECS
**S10**	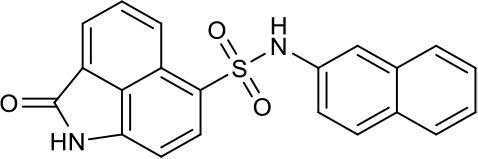	19.1 ± 2.2	SPECS
**S21**	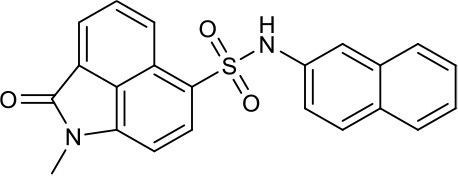	28.8 ± 3.1	SPECS
**S22**	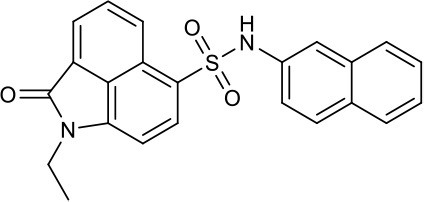	16.0 ± 1.8	SPECS
**S23**	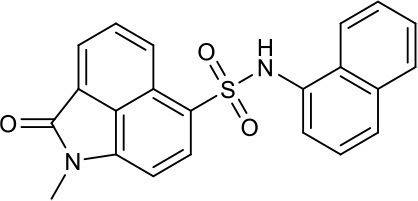	24.6 ± 2.1	SPECS
**S24**	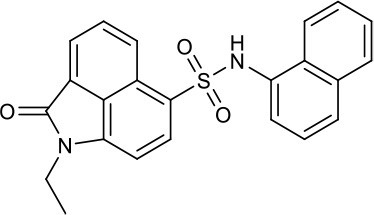	19.8 ± 1.5	SPECS
**S25**	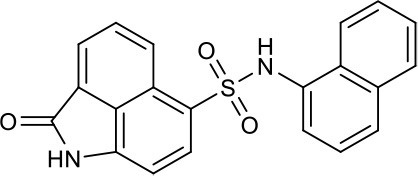	14.0 ± 2.3	SPECS
**S26**	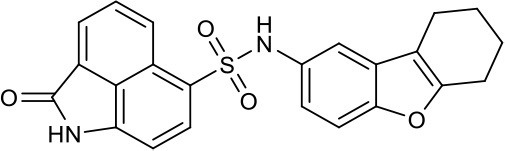	16.0 ± 2.3	SPECS
**S27**	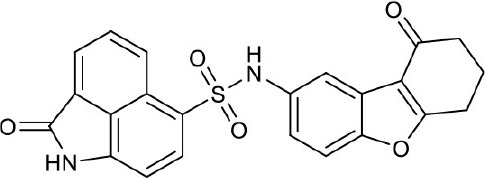	28.5 ± 3.8	SPECS
**4a**	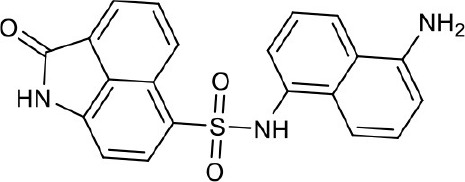	>100	Synthesis
**4b**	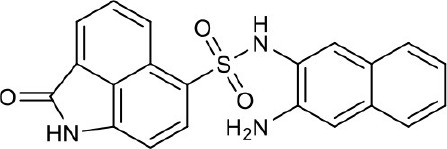	>100	Synthesis
**4c**	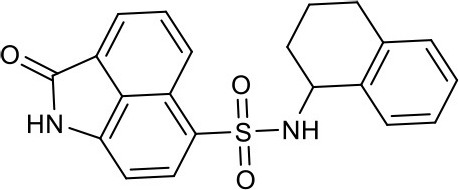	12.5 ± 1.6	Synthesis
**4d**	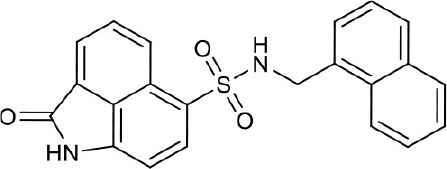	>100	Synthesis
**4e**	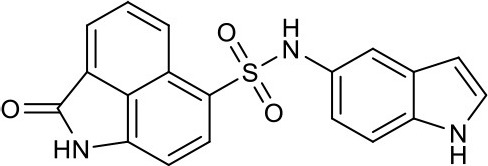	3.0 ± 0.8	Synthesis
**4f**	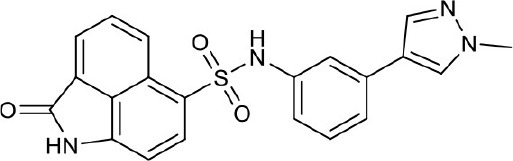	6.2 ± 1.3	Synthesis
**4g**	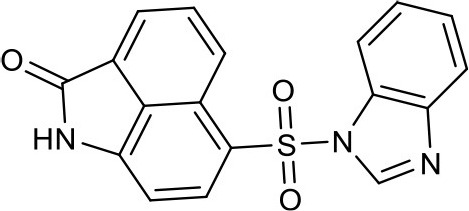	>100	Synthesis
**SPD304**	6.4 ± 0.6		

a *Data shown represent the mean (n = 3)*.

All the compounds were tested using the TNF-α induced NF-κB reporter assay. The structures and activities were listed in Table 1. For **S10**, methyl or ethyl group substitution on the amide of 2-oxo-1,2-dihydrobenzo[*cd*]indole-6-sulfonamide had no obviously enhanced inhibition (**S21** and **S22**), and the α or β substitution of the naphthyl group did not affect the inhibition (**S23, S24**, and **S25**). The size of the N-substituted groups of sulfonamide was important for inhibition activity (**EJMC-1, S10**, and **S27**). The flexibility and aromaticity of the N-substituted two-ring group of sulfonamide played dominant role, too rigid or too flexible dramatically reduced the activity (**4g**, **4d**). The fact that N-(5-aminonaphthalen-1-yl) and N-(3-aminonaphthalen-1-yl) group substituted compounds lost functions might be caused by the conformation change due to additional amino group on the naphthalene ring. Introducing heterocycle significantly increases the inhibition activity (**4e and 4f**). The N-(*1H*-indol-6-yl) substituted sulfonamides (**4e**) were 6-fold more potent than **S10**, even better than SPD304 (Table 1, Figure 3B). Though **S10** and **4e** had similar size of substitution group on sulfonamide, **4e** shown better inhibition activity than **S10** might due to the additional H-bond that **4e** forms with the backbone carbonyl of Gly121 (Figure 4C). Meanwhile, the indolyl group of **4e** was also deeper in the binding pocket than that of naphthyl group on **S10** (Figure 4D).

## Conclusion

We have optimized a previously reported TNF-α inhibitor **EJMC-1** using similarity-based VS and rational design. An analog of **EJMC-1**, **S10** was found with 2-fold TNF-α increased inhibition activity. Based on the structures of **EJMC-1**, **S10**, and their interactions with TNF-α, we designed derivatives of 2-oxo-1,2-dihydrobenzo[*cd*]indole-6-sulfonamide. Several commercially available ones were purchased and seven new compounds were synthesized for SAR study. After two rounds of design, we obtained **4e** with an IC_50_ of 3.0 ± 0.8 μM, which is one of the most potent TNF-α small molecule inhibitors reported so far. Compound **4e** provides a good starting point for developing more potent TNF-α small molecule inhibitors.

## Author contributions

LL and YL designed and guided this study; XD designed the research, performed molecular docking and similarity search, and conducted the chemical synthesis; XZ performed the cell assay; HL and QS participated in the cell assay; BT performed the SPR binding assay; XD, LL, and YL analyzed the data and wrote the manuscript with input from all authors; XD and XZ have equal contribution of this work.

### Conflict of interest statement

The authors declare that the research was conducted in the absence of any commercial or financial relationships that could be construed as a potential conflict of interest.
