# Supplementary material for: Design, Synthesis, and Evaluation of Dihydrobenzo[cd]indole-6-sulfonamide as TNF-α Inhibitors
Source: Front Chem. 2018 Apr 4;6:98. doi: 10.3389/fchem.2018.00098 (PMC5893771; doi:10.3389/fchem.2018.00098)
Supplement: Supplementary file 1 [file Table1.PDF]

Supplementary Table S1

Table S1. Screened compounds

Compounds	Structure	SPECS ID	SPR competitive
S1	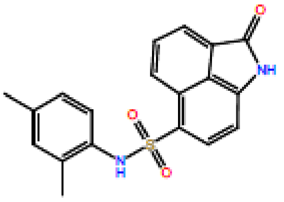	AQ-390/10775029	Yes
S2	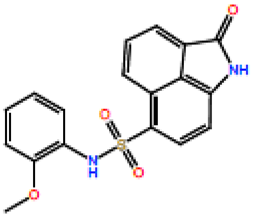	AG-690/10773058	Yes
S3	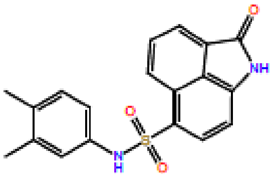	AQ-390/10775033	Yes
S4	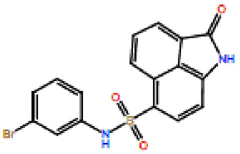	AQ-390/10770014	Yes
S5	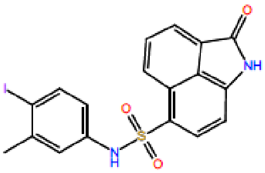	AQ-390/10773037	Yes
S6	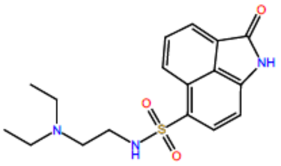	AG-690/10769052	Yes

S7	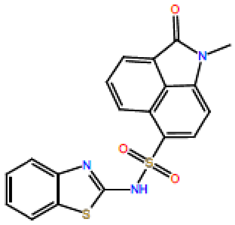	AQ-390/43364041	Yes
S8	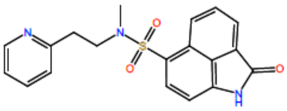	AQ-390/43238272	Yes
S9	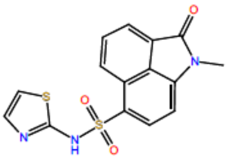	AQ-390/43364057	Yes
S10	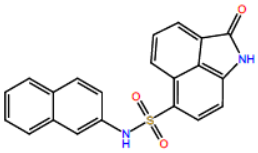	AG-690/10781009	Yes
S11	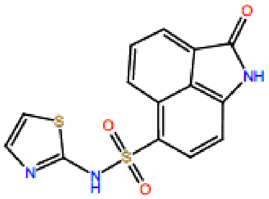	AQ-390/43364066	Yes
S12	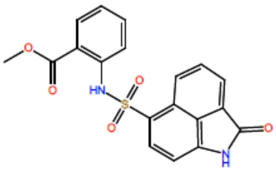	AG-690/10776038	Yes
S13	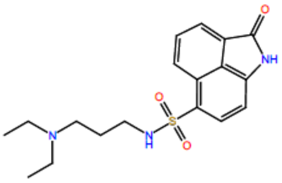	AQ-390/43364049	Yes

S14	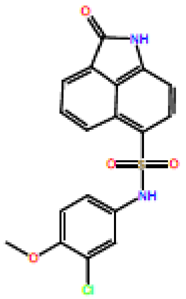	AQ-390/43238270	Yes
S15	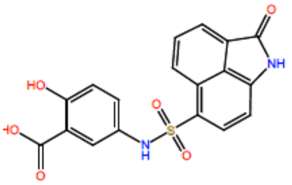	AG-690/10775008	Yes
S16	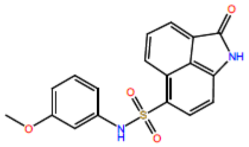	AG-690/10773059	Yes
S17	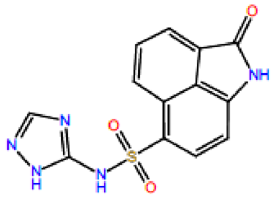	AG-690/10758045	Yes
S18	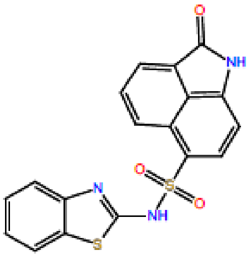	AG-690/10783048	Yes
S19	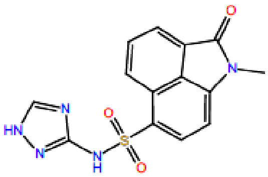	AQ-390/43364010	Yes

S20	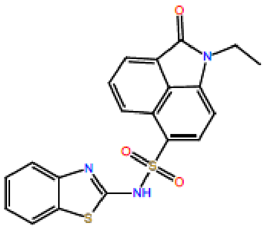	AQ-390/40910467	Yes
21	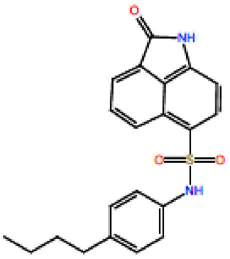	AQ-390/10779040	No
22	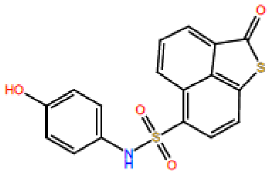	AQ-390/41004228	No
23	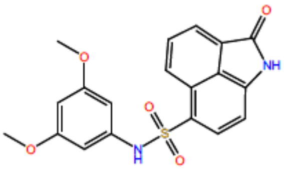	AG-690/10776061	No
24	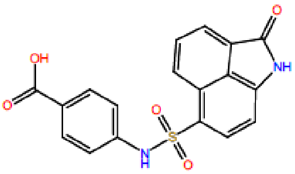	AG-690/10774038	No
25	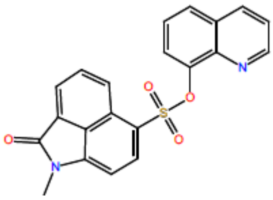	AQ-390/42869041	No
26	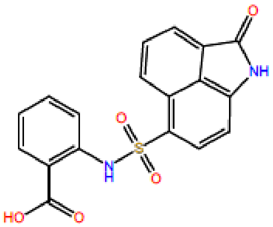	AG-690/10774036	No

27	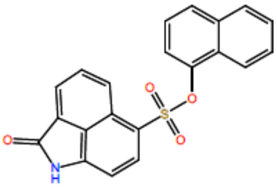	AG-690/15438104	No
28	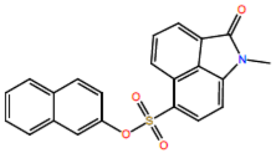	AQ-390/42869039	No
29	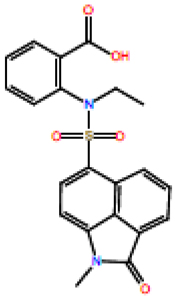	AQ-390/43364050	No
30	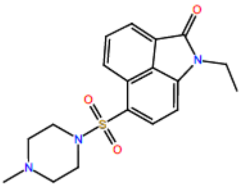	AF-399/12151046	No
31	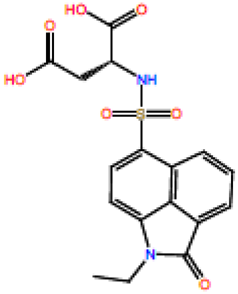	AQ-390/43364020	No
32	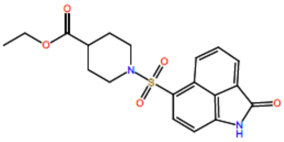	AQ-390/43238175	No
33	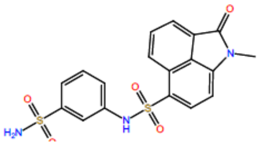	AQ-390/43363962	No

34	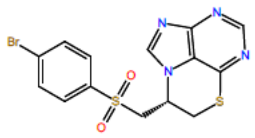	AP-853/43464237	No
35	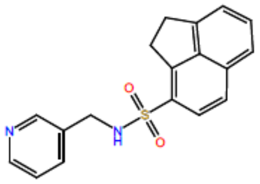	AP-263/43028001	No
36	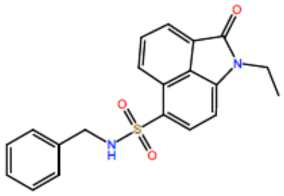	AQ-390/40910322	No
37	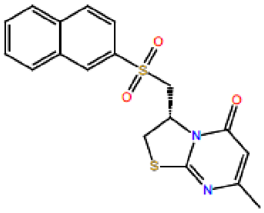	AP-853/43416457	No
38	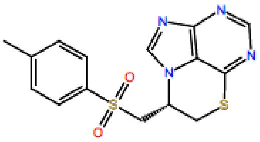	AP-853/42402795	No
39	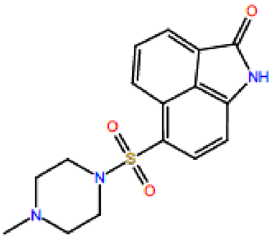	AQ-390/43238267	No
40	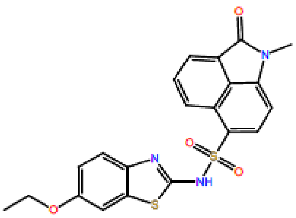	AQ-390/42869137	No

41	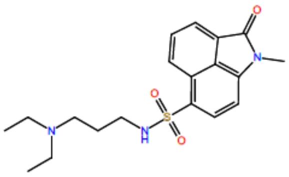	AQ-390/43364015	No
42	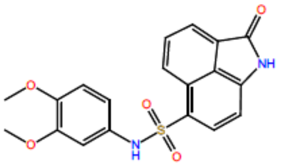	AG-690/10758030	No
43	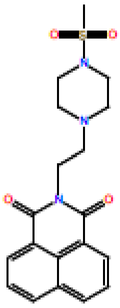	AF-399/40826447	No
44	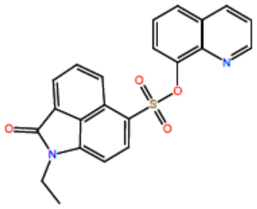	AQ-390/42869040	No
45	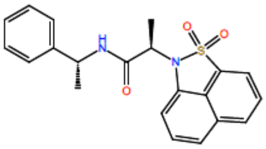	AO-081/42858858	No
46	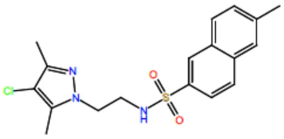	AP-124/43383405	No
47	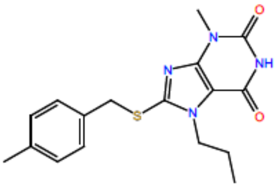	AG-690/13701134	No

48	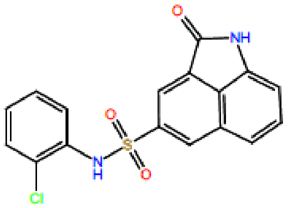	AQ-390/42709102	No
49	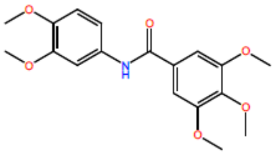	AO-548/11812423	No
50	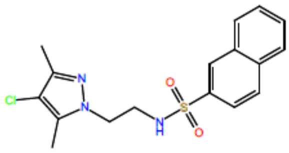	AP-124/43383404	No
51	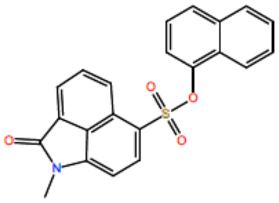	AQ-390/42869036	No
52	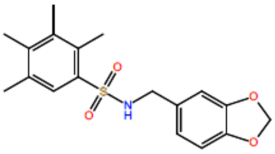	AP-263/43302399	No
53	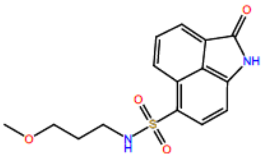	AG-690/10769010	No
54	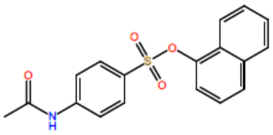	AN-698/40847879	No

55	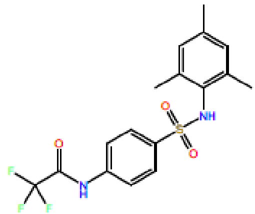	AN-329/40869084	No
56	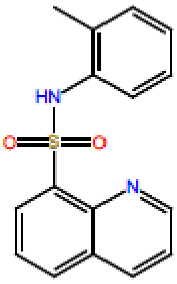	AQ-390/11990646	No
57	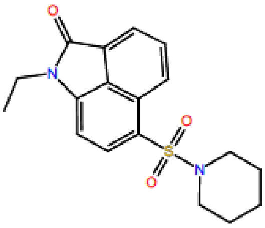	AF-399/12151063	No
58	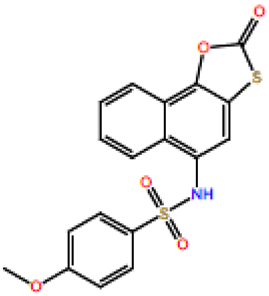	AP-906/41640278	No
59	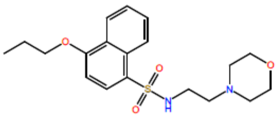	AP-263/43241435	No
60	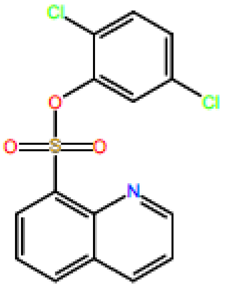	AQ-390/42122907	No

61	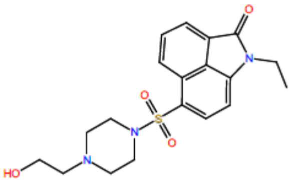	AF-399/13199001	No
62	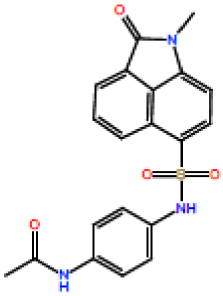	AQ-390/42869112	No
63	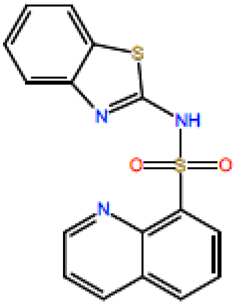	AQ-390/10476009	No
64	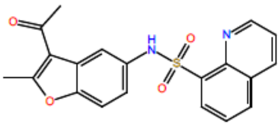	AQ-390/42708547	No
65	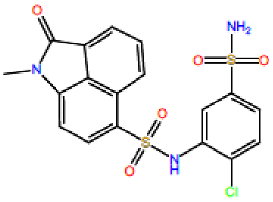	AQ-390/43363959	No
66	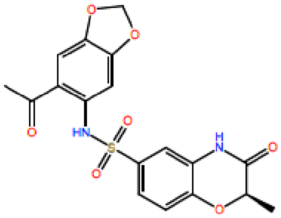	AO-080/43441830	No

67	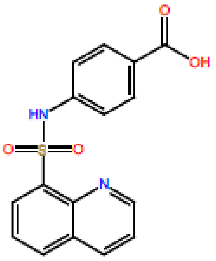	AE-641/12753614	No
68	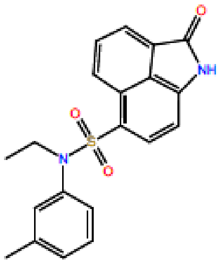	AQ-390/10779024	No
