# Supplementary material for: Design, Synthesis, and Evaluation of Dihydrobenzo[cd]indole-6-sulfonamide as TNF-α Inhibitors
Source: Front Chem. 2018 Apr 4;6:98. doi: 10.3389/fchem.2018.00098 (PMC5893771; doi:10.3389/fchem.2018.00098)
Supplement: Supplementary file 2 [file Table2.PDF]

Table S2. Screened compounds by cell assay

Compounds	Inhibition rate at 62.5 μM[a]	SD	Inhibition rate at 20.0 μM[a]	SD
S1	46.64	1.93	46.64	1.57
S2	31.18	3.00	29.81	1.91
S3	79.27	1.41	52.24	2.11
S4	<5	—	<5	—
S5	64.91	0.65	34.68	1.75
S6	25.72	2.56	24.19	4.66
S7	<5		<5	
S8	25.61	6.77	23.27	3.19
S9	<5	—	<5	—
S10	99.49	0.09	59.71	3.28
S11	<5	—	<5	—
S12	34.83	2.63	38.40	2.70
S13	20.91	1.26	6.38	3.81
S14	30.32	7.26	40.40	2.68
S15	26.96	2.60	26.43	2.24
S16	29.14	2.53	31.55	2.01
S17	14.58	4.27	22.30	2.72
S18	<5	—	<5	—
S19	15.41	6.04	15.38	3.21
S20	<5	—	<5	—
EJMC-1	74.78	1.63	50.19	1.88

[a] Data shown represent the mean (n = 3).
