# Supplementary material for: Design, Synthesis, and Evaluation of Dihydrobenzo[cd]indole-6-sulfonamide as TNF-α Inhibitors
Source: Front Chem. 2018 Apr 4;6:98. doi: 10.3389/fchem.2018.00098 (PMC5893771; doi:10.3389/fchem.2018.00098)
Supplement: Supplementary file 3 [file Table3.PDF]

Table S3. SPECS ID of **S10** analogues

compounds	SPECS ID
S21	AQ-390/43364045
S22	AQ-390/43363994
S23	AQ-390/42869139
S24	AQ-390/42869140
S25	AQ-390/10781006
S26	AG-690/15438056
S27	AG-690/15438055
