# Supplementary material for: Design, Synthesis, and Evaluation of Dihydrobenzo[cd]indole-6-sulfonamide as TNF-α Inhibitors
Source: Front Chem. 2018 Apr 4;6:98. doi: 10.3389/fchem.2018.00098 (PMC5893771; doi:10.3389/fchem.2018.00098)

Design, Synthesis, and Evaluation of Dihydrobenzo[*cd*]indole-6-sulfonamide as TNF-α Inhibitors

Xiaobing Deng[1, 2], Xiaoling Zhang[2], Bo Tang[3], Hongbo Liu[1], Qi Shen[2], Ying Liu[2, 3*],

Luhua Lai[1, 2, 3*]

[1] *Peking−Tsinghua Center for Life Sciences, Peking University, Beijing 100871, China.* [2] *Center for Quantitative Biology, Academy for Advanced Interdisciplinary Studies, Peking University, Beijing 100871, China.* [3] *BNLMS, State Key Laboratory for Structural Chemistry of Unstable and Stable Species, College of Chemistry and Molecular Engineering, Peking University, Beijing 100871, China*

**Correspondence:* Ying Liu, liuying@pku.edu.cn

Luhua Lai, lhlai@pku.edu.cn

Supplementary spectra of featured compounds

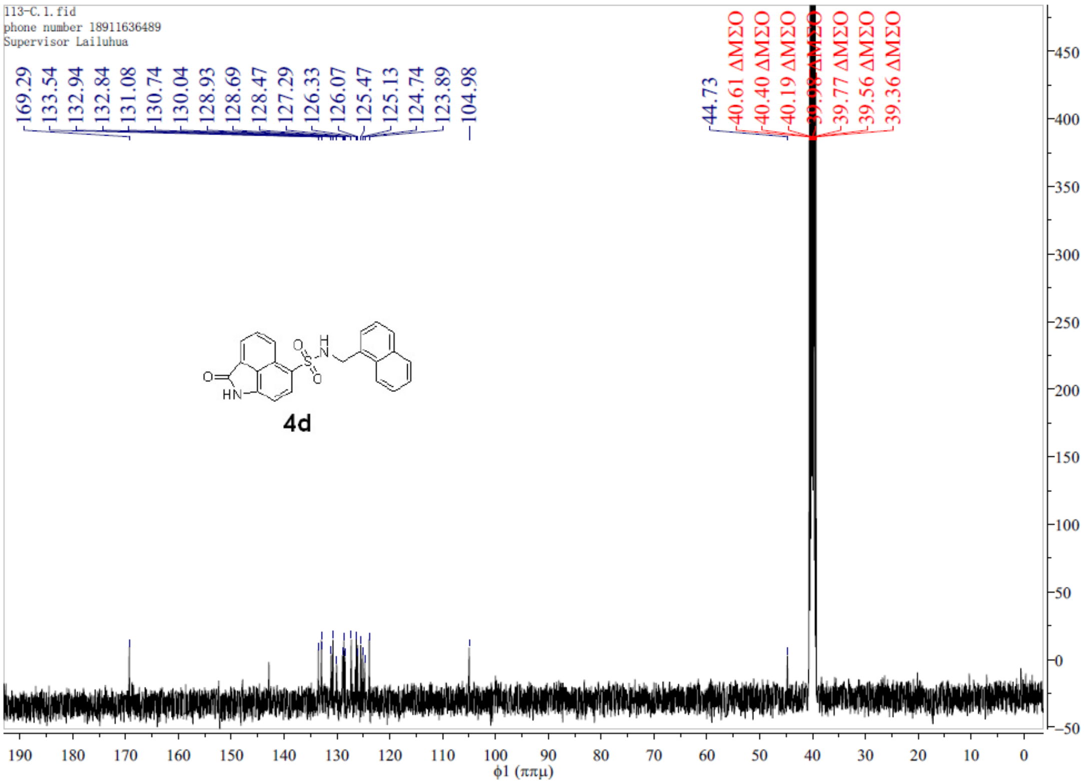
113-C.1.fid
phone number 18911636489
Supervisor Lailuhua
169.29
133.54
132.94
132.84
131.08
130.74
130.04
128.93
128.69
128.47
127.29
126.33
126.07
125.47
125.13
124.74
123.89
104.98
44.73
40.61 ΔΜΣΟ
40.40 ΔΜΣΟ
40.19 ΔΜΣΟ
39.98 ΔΜΣΟ
39.77 ΔΜΣΟ
39.56 ΔΜΣΟ
39.36 ΔΜΣΟ
4d
φ1 (ππμ)

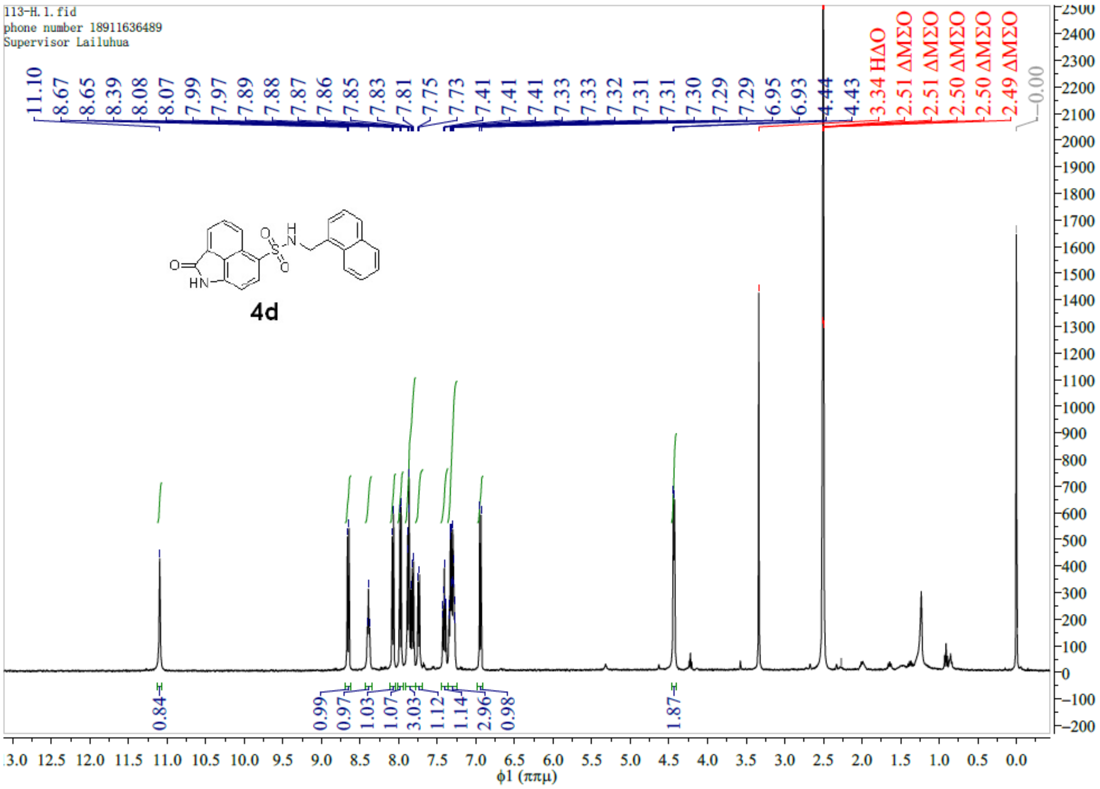
113-H.1.fid
phone number 18911636489
Supervisor Lailuhua
11.10
8.67
8.65
8.39
8.08
8.07
7.99
7.97
7.89
7.88
7.87
7.86
7.85
7.83
7.81
7.75
7.73
7.41
7.41
7.41
7.33
7.33
7.32
7.31
7.31
7.30
7.29
7.29
6.95
6.93
4.44
4.43
3.34 ΗΔΟ
2.51 ΔΜΣΟ
2.51 ΔΜΣΟ
2.50 ΔΜΣΟ
2.50 ΔΜΣΟ
2.49 ΔΜΣΟ
0.00
4d
0.84
0.99
0.97
1.03
1.07
3.03
1.12
1.14
2.96
0.98
1.87
φ1 (ππμ)

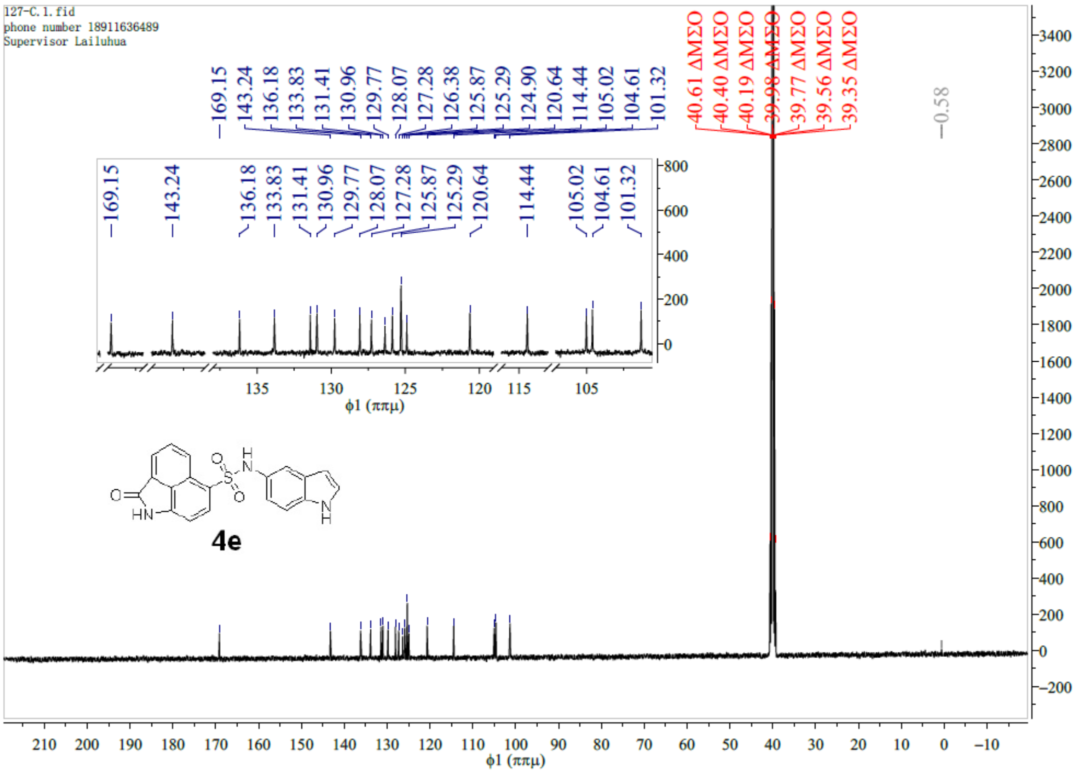
127-C.1.fid
phone number 18911636489
Supervisor Lailuhua
169.15
143.24
136.18
133.83
131.41
130.96
129.77
128.07
127.28
126.38
125.87
125.29
124.90
120.64
114.44
105.02
104.61
101.32
40.61 ΔΜΣΟ
40.40 ΔΜΣΟ
40.19 ΔΜΣΟ
39.98 ΔΜΣΟ
39.77 ΔΜΣΟ
39.56 ΔΜΣΟ
39.35 ΔΜΣΟ
-0.58
φ1 (ππμ)
4e

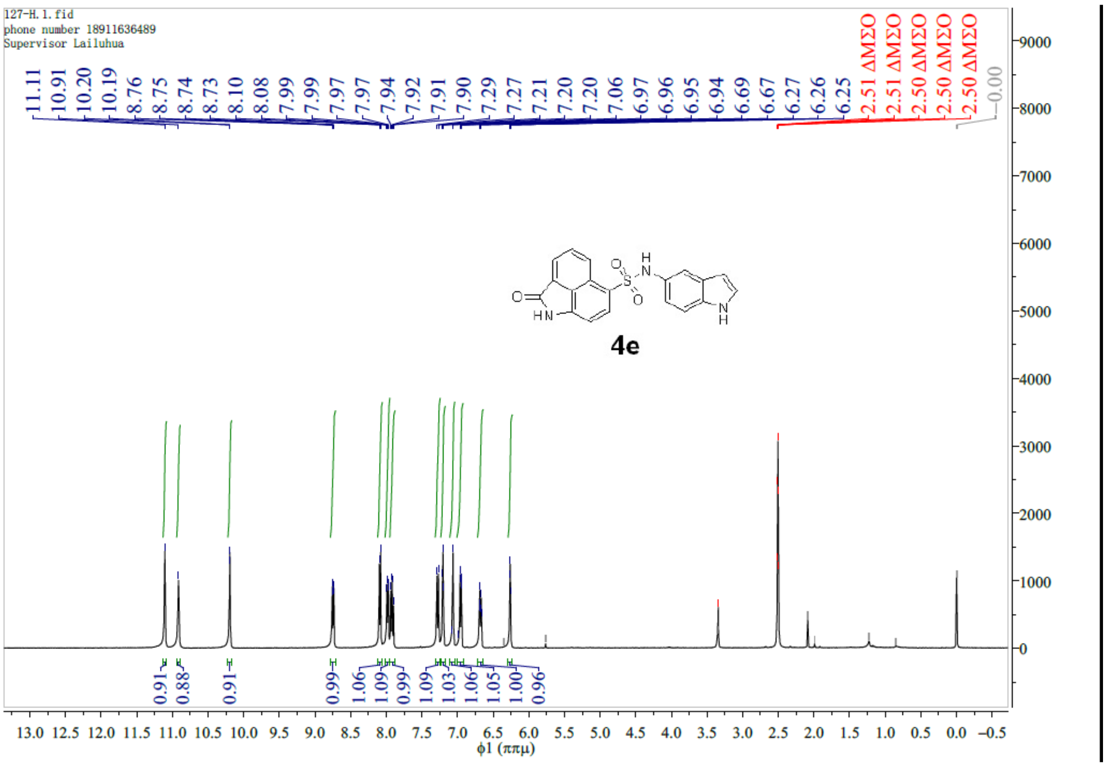
127-H.1.fid
phone number 18911636489
Supervisor Lailuhua
11.11
10.91
10.20
10.19
8.76
8.75
8.74
8.73
8.10
8.08
7.99
7.99
7.97
7.97
7.94
7.92
7.91
7.90
7.29
7.27
7.21
7.20
7.20
7.06
6.97
6.96
6.95
6.94
6.69
6.67
6.27
6.26
6.25
2.51 ΔΜΣΟ
2.51 ΔΜΣΟ
2.50 ΔΜΣΟ
2.50 ΔΜΣΟ
2.50 ΔΜΣΟ
0.00
0.91
0.88
0.91
0.99
1.06
1.09
0.99
1.09
1.03
1.06
1.05
1.00
0.96
φ1 (ππμ)
4e

HRMS for compound **4e.**

Peking University Mass Spectrometry Sample Analysis Report

Analysis Info

Analysis Name	FTMS-18010060_Pos_20180108_000001.d	Acquisition Date	1/8/2018 11:50:39 AM
Sample	36	Instrument	Bruker Solarix XR FTMS
Comment		Operator	Peking University

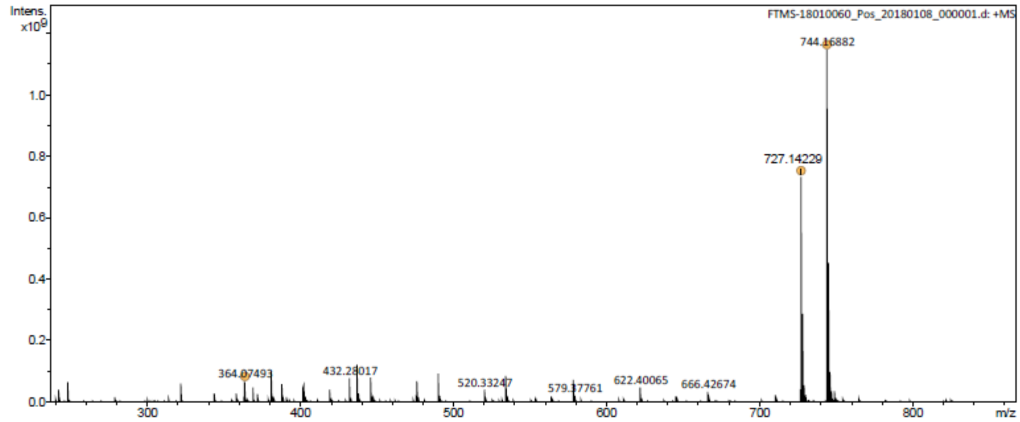

Meas. m/z	#	Ion Formula	Score	m/z	err [ppm]	Mean err [ppm]	mSigma	rdb	e⁻ Conf	N-Rule
364.074934	1	C19H14N3O3S	100.00	364.075039	0.3	-0.1	7.3	15.0	even	ok
727.142293	1	C38H27N6O6S2	100.00	727.142801	0.7	-0.4	64.9	29.0	even	ok
744.168819	1	C38H30N7O6S2	100.00	744.169350	0.7	2.2	65.0	28.0	even	ok

HRMS for compound **4d.**

Peking University Mass Spectrometry Analysis Report

Analysis Info

Analysis Name	FTMS-18010042_Pos_20180105_000007.d	Acquisition Date	1/5/2018 3:14:00 PM
Sample	113	Instrument	Bruker Solarix XR FTMS
Comment		Operator	Peking University

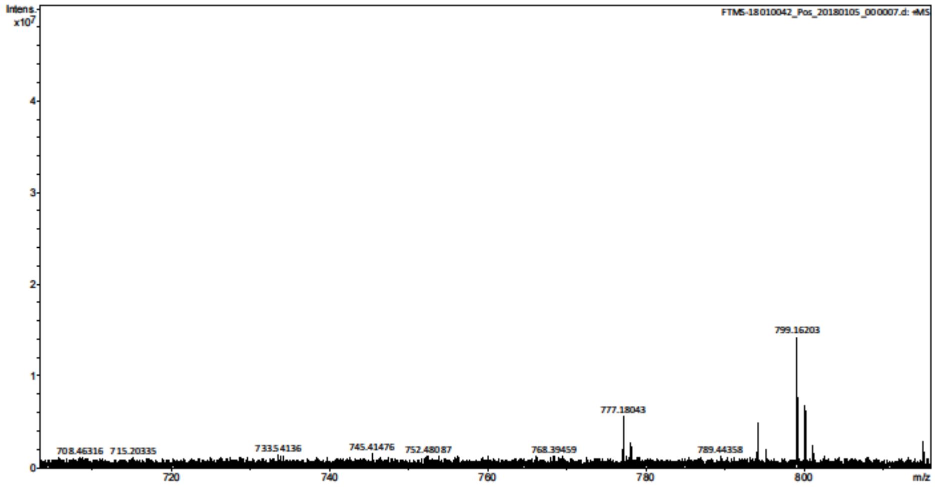

Supplement: Supplementary file 4 [file Image1.pdf]
